# Cod Liver Oil’s Encapsulation into Sodium Alginate/Lupin Protein Beads and Its Application in Functional Meatballs’ Preparation

**DOI:** 10.3390/foods11091328

**Published:** 2022-05-03

**Authors:** Essam Mohamed Elsebaie, Mona Morgan Kassem, Mona Metwally Mousa, Mahmoud Abdelhakiem Mansour Basuony, Neveen M. Zeima, Rowida Younis Essa

**Affiliations:** 1 Food Technology Department, Faculty of Agricultural, Kafrelsheikh University, Kafr El-sheikh 33516, Egypt; rowida.eisa@agr.kfs.edu.eg; 2 Agricultural Engineering Department, Faculty of Agricultural, Kafrelsheikh University, Kafr El-sheikh 33516, Egypt; Mona2Kassem@gmail.com (M.M.K.); maqbdba42@gmail.com (M.A.M.B.); 3 Food Science & Technology Department, Faculty of Home Economics, Al-Azhar University, Tanta 11651, Egypt; baraahmebara@gmail.com; 4 Nutrition & Food Science Department, Faculty of Home Economics, Al-Azhar University, Tanta 11651, Egypt; Nevmzei42@gmail.com

**Keywords:** cod liver oil, fortification, meatballs, alginate/lupine protein

## Abstract

Cod liver oil (CLO) is an essential source of healthy ω-3 fatty acids to be employed in functional meals. However, its autoxidation sensitivity, solubility, and odour present it as challenging to handle. Its encapsulation might mitigate these problems. This research studied using alginate/lupine protein as a wall material for CLO encapsulation as well as to characterise CLO microcapsules for their size, sphericity factor, encapsulation efficiency, morphology (scanning electron microscopy), in vitro release, and thermal stability. In this study, the oxidative stability, quality parameters, and sensory attributes of meatballs enriched with free CLOs and encapsulated CLOs throughout storage at 4 ± 1 °C for 16 days were assessed. The CLO microspheres had a homogeneous round shape, a diameter of 0.82 ± 0.06 mm, a sphericity factor of 0.092 ± 0.01, an encapsulation efficiency of 95.62% ± 1.13%, and an accumulative release rate of 87.10% after 270 min in the stimulated gastrointestinal conditions. Additionally, it was discovered that encapsulated oil was more stable than free CLOs to heat treatments (70–100 °C, 24 h). pH, thiobarbituric acid-reactive substances, peroxide value, conjugated dienes value, and carbonyl content of meatballs enriched with microencapsulated CLOs were significantly lower when compared to free CLOs and/or control samples. CLO microcapsules improved the sensory characteristics of meatballs throughout storage; however, meatballs directly containing CLOs were rejected. Thus, the viability of alginate/LPI complex microcapsules containing CLOs to enrich meat products subjected to storage with refrigeration could be indicated without any change in the characteristics.

## 1. Introduction

Meatballs are considered as a popular meat product that are made from lower-quality trimmed red meat to create another meat product with higher quality [1]. They are high in protein, minerals, lipids, vitamins, and vital amino acids, along with being readily available, inexpensive, and ready to eat [2]. Consumption of ready-to-eat meals, also known as “quick meals,” is on the rise around the world as a result of a more hectic modern lifestyle [3]. One of these “quick meals” is the meatball, which is made from a combination of 53% minced lean beef, 17% fat, and a few salts (phosphates, monosodium glutamate, sodium chloride), subsequently shaped into the proper form and frozen until cooking [4].

CLOs have generated tremendous interest in the food industry as a nutritional additive, owing to their high content of omega-3 fatty acids, specifically docosahexaenoic acid and eicosapentaenoic acid [5]. In addition, compared with fish oil, CLO has more vitamins D and A [6]. CLOs have been linked to a number of health benefits, including anti-diabetic, anti-inflammatory, newborn rickets prevention, anticancer, and cardiovascular disease risk reduction [7,8,9,10].

Supplementing food items with fish oil may help to encourage a higher level of omega-3 intake in countries where fish consumption is low [11]. As bioactive chemicals have a key role in the protection from certain illnesses and the promotion of health, food supplementation with polyunsaturated fatty acids from marine sources is an important topic [12]. A viable strategy for increasing omega-3 fatty acid intake and creating a healthy food for concerned customers might be to enrich these foods with fish oil to meet the recommended daily allowance of these fatty acids (80 mg/100 g) [13].

Despite all of the advantages of omega-3 fatty acids, among the most problematic aspects of enriching with them is their great sensitivity to oxidation [14], which causes the formation of poisonous or unattractive off-flavour components and gives the oil a distinct fragrance, which makes it hard to utilise [11]. Encapsulation methods are another option for overcoming these obstacles [15].

Encapsulation is the process of enclosing active components in a particulate matrix in order to accomplish one or more desired impacts [16]. Encapsulation of oils is performed for a variety of purposes, including converting liquids to solids for easier handling, transport, and integration into other constituents. Other considerations include masking taste and odour, preventing evaporation or oxidation, and implementing controlled-release mechanisms [17]. Though emulsion spray-drying is the most often used method for oil encapsulations, using a high temperature might cause the oils to degrade prematurely [18].

Ionic gelation-produced hydrogels have been utilised to microencapsulate bioactive substances. Alginate particles are formed by a chemical reaction between a polymer and divalent or trivalent ions, such as calcium, which results in a tri-dimensional matrix. This technology allows for the creation of high-oil-content particles that may be used to enhance foods and generate functional food products [19].

Due to its high solubility and nontoxicity, sodium alginate is commonly utilised throughout the ionic gelation process [20]. Despite the benefits of ionic gelation for microencapsulation, sodium alginate matrices have certain drawbacks due to their porous structure, which may expose the encapsulated component to the environment and induce deterioration. To enhance hydrogel characteristics, researchers have employed several substances as fillers into the alginate matrix, such as polysaccharides and proteins [21].

Since ancient times, lupine seeds have been utilised as a protein source [22]. Nowadays, there is a growing interest in expanding the use of this legume seed [23]. This is mostly owing to its similarities to soybeans as a rich protein source, as well as the reality that it can be cultivated in a larger climatic range [24]. Furthermore, its ability to adapt to poor (i.e., leachable) soils makes it commercially viable. Lupine is often used in snacks in the Middle East and is becoming popular in other areas of the world as a high-protein soy alternative [25]. El-Adawy et al. [26] and Piornos et al. [27] claim that a protein isolate derived from lupin seed powder is mainly composed of high molecular weight proteins α- and β-conglutins (between 39 and 69 kDa), and it exhibits high emulsifying and foaming capabilities. The study of Piornos et al. [28] is the only investigation about using lupine protein in combination with alginate as a shell material during oil encapsulation processes. As a result, it has been intriguing to investigate the impact of these proteins on the stability and effectiveness of oil encapsulation into beads. Despite the fact that bulk fish oil enrichment has been tested, the literature on food enriched with microencapsulated omega-3 polyunsaturated fatty acids is limited, with only a few lactic products [29,30], bakery products [31], beverages [32,33], and meat products [34,35]. Hence, the ongoing study concentrated on two major goals. The first one is to use alginate/lupine protein as a wall material for CLO encapsulation as well as to characterise CLO microcapsules for their size, sphericity factor, encapsulation efficiency, morphology (scanning electron microscopy), in vitro release, and thermal stability (Rancimat). The second one is to produce functional meatballs enriched with free CLOs and encapsulated CLOs and to assess their oxidative stability, quality parameters, and sensory attributes throughout cold storage at 4 ± 1 °C for 16 days.

## 2. Materials and Methods

### 2.1. Materials

Seeds of lupine (Lupinusalbus L. variety Giza) were received from the Giza Agricultural Research Centre in Egypt. All of the components for the meatballs were obtained from a local supermarket in Kafr El-Shiekh, Egypt. Sigma-Aldrich Chemical Co. provided cod liver oils (CLOs) and sodium alginate (MW 20–40 kDa) (St. Louis, MO, USA). El-Naser Chemical Co. provided the calcium chloride (CaCl2) (Cairo, Egypt).

### 2.2. Methods

#### 2.2.1. Lupine Protein Isolate (LPI) Preparation

Lupine seeds were de-hulled before being pulverised in an electrical mill to produce a fine powder (650 µm particle size) and stored at −20 °C until use. LPI was produced using the solubilisation-isoelectric/precipitating technique reported by Kalaydzhiev et al. [36]. First, a 1:10 (w/v) mixture of lupin flour and distilled water was prepared. The mixture pH value was changed to 9.0 by adding 1 M NaOH and stirring for 90 min at 25 °C. The mixture was then centrifuged at 3300× *g* for 10 min. The isolated proteins were recovered from the supernatant. The supernatant was then adjusted to pH 4.5 with 1 M HCl for protein precipitation at the isoelectric point. Proteins were then collected by centrifugation at 3300× *g* for 15 min. The obtained protein was lyophilised immediately after production and kept at 4 °C until it was used.

#### 2.2.2. CLO Microencapsulation

##### Emulsion Preparation

The polymer-CLOs emulsion was prepared according to the methodology proposed by Volić et al. [37]. Firstly, 2.8 g of LPI was hydrated in 50 mL of distilled water at room temperature, and the resulting dispersion was adjusted to pH 9.0 with NaOH (1 mol/L). After 90 min of stirring, the pH was corrected to 7.0 using HCl (1 M). Separately, 2.35 g of sodium alginate was dispersed in 50 mL of distilled water to make a solution. This solution was left at room temperature for at least 3 h with regular stirring, until the alginate was completely dissolved. After combining the two prepared solutions, CLOs were added to the final alginate/LPI solution at a weight ratio of 15:100 (oil phase:water phase) and emulsified for 3 min using an UltraTurrax homogeniser (UltraTurrax IKA T18) at 12,000 rpm.

##### Beads’ Preparation

The preparation of alginate beads was conducted following the technique outlined by Volić et al. [37]. Ten millilitres of mix solutions were dripped into 100 mL of CaCl_2_ (1.5%) using a 20 G flat-tipped hypodermic needle at a distance of 12 cm. The beads were allowed to solidify in the CaCl_2_ solution for 30 min. The beads were then acquired using vacuum filtering and dried in a 45 °C oven until they reached a consistent weight. Finally, the glassy beads were wrapped in polyethylene bags and preserved in the desiccator until they could be analysed further.

#### 2.2.3. Characterisation of Microspheres

##### Size Determination and Sphericity Factor (SF)

Beads’ images were taken in order to determine their shape and size. The size of the beads and their SF were determined using a digital light microscope (Motic, BA210, Motic Inc., BC, Canada) and image analysis software (Motic Images Plus 2.1 ML, Motic Inc., BC, Canada). SF was determined using the equation by Chan et al. [38]:

SF = (d_max_ − d_min_)/(d_max_ + d_min_) (1)

where d_max_ is the maximum bead diameter and d_min_ is the minimum diameter.

##### Encapsulation Efficiency (EE)

With minor modifications, the EE of CLOs was measured using the approach published by Volić et al. [37]. Beads were first pulverised with a hammer and mortar. In the mortar, a sheet of filter paper was inserted to collect any oil that was emitted during the grinding process. CLOs were extracted from ground beads and filter paper using three solvents: diethyl ether, ethanol, and petroleum ether, in that order (50 mL for each). The solvents were evaporated using a rotary evaporator (Stuart RE402, United Kingdom) at 100 rpms/15–30 min at 35, 79, and 40 °C, respectively. The resulting solution (oil extract) was filtered using Whatman No. 1 filter paper soaked in anhydrous Na_2_SO_4_, and the extracted oil was dried to a consistent weight. The encapsulation efficiency was determined by dividing the percentage of encapsulated oil (superficial and inner oil) after drying by the quantity of oil utilised in the encapsulation test.

##### Microscopic Observations

Prior to the examination, beads were coated using JFC1100E Sputter Coater equipment, JFC1100E (JOEL Ltd., Tokyo, Japan), using gold/platinum alloy (15/85). After the deposition of a thin gold/palladium covering, the beads were observed by a JOEL6360LA (JEOL Ltd., Tokyo, Japan) scanning electron microscope, at an acceleration voltage of 20 kV.

##### In Vitro Release

The method of Liu et al. [39] was used to assess the in vitro release of CLOs from microcapsules beneath simulated gastrointestinal conditions. Gastric and enteric digesting solutions were produced, following the method of Bouayed et al. [40]. Alginate–LPI beads with encapsulated CLOs (10 g) were submerged in 10 mL of gastric fluid (SGF) and incubated for 2 h at 37 °C/100 rpm. To maintain a constant volume across time intervals, about 2 mL of the specimens was taken and replaced with new medium. Immediately after 2 h, the microcapsules were collected and submerged in 10 mL of simulated intestinal solution (SIF) and incubated for 3 h at 37 °C/100 rpm. To maintain a constant volume over time intervals, about 2 mL of the samples was extracted and replaced with new medium. The CLO content of the specimens was tested in the same manner as indicated for EE. The experiment was carried out three times.

##### Thermal Stability

The microcapsules were subjected to an accelerated oxidation experiment in a Rancimat (Metrohm 743, Herisau, Switzerland) apparatus at high temperatures (80 and 100 °C), with a continual bubble of airflow at 20 L/h. CLOs that had not been encapsulated were used as a control [14]. For each replication, measurements were performed in at least two different ways.

#### 2.2.4. Meatballs’ Manufacture

According to Essa and Mostafa [2], meatballs were made with 75% red lean beef, 25% back fat, and 1.5% sodium chloride. All of the components were combined for 7 min in a bowl mixer before being separated into three groups. The first group (control) without CLOs, the second group (T1: CLOs direct addition), and the third group (T2: encapsulated CLOs). Meatballs (302 g) were shaped by hand into a spherical shape. The meatballs were kept for 16 days at 4 ± 1 °C in cardboard cartons with a plastic liner. Following that, the meatball samples were cooked in water for 20 min at 80 °C. Before analysis, all samples were boiled, drained, and stored in plastic bags. After 0, 4, 8, 12, and 16 days of storage, specimens were obtained.

##### Physicochemical Properties of Meatballs

pH measurement:

Specimens of 3 g were homogenised in 27 mL of miliQ water. A pH meter (HAANA, HI902 meter, Weilheim, Germany) was used to test the pH of homogenised specimens.

Thiobarbituric acid-reactive substances (TBARS):

TBARS levels were measured in milligrams of malonaldehyde (MDA) per kilogram of specimen using the spectrophotometric technique of Yoon et al. [41]. Five-grams of minced meatballs was combined with 25 mL of TBA stock reagent (0.034 g TBA, 15% trichloroacetic acid solution) and heated (95 °C for ten minutes). Subsequent samples were chilled under running tap water. The samples were then centrifuged (5600 rpm, 20 min), the absorbance (532 nm) was measured, and the samples’ TBARS value was calculated using a standard curve.

Peroxide value:

Total lipid extract of meatballs was prepared by soaking minced meatballs in Folch solution using the ratio 1:5 (w/v). Then, meatball peroxide values (PVs) were estimated in the lipid extract using the titration procedure outlined by Shantha and Decker [42]. The findings were presented in milliequivalents of peroxide/kg fat.

Conjugated dienes:

Conjugated dienes content was estimated using the procedure of Juntachote et al. [43]. Initially, 1 g of meatballs was homogenised in 10 mL of miliQ water. The suspension (0.5 mL) was then mixed with 5 mL of extraction solution (hexane/isopropanol, 3:1 (v/v)). The mixture was centrifuged at 3500 rpm for 5 min, and the absorbance at 233 nm was determined. The quantity of conjugated dienes in the specimen was calculated as mole/mg using the molar extinction value of 25,200 M^−1^ cm^−1^.

Carbonyl content:

Meatballs’ carbonyl content was analysed using the technique of Elsebaie et al. [44]. In summary, 1 g of meatball was mashed for 1 min in 20 mM of sodium phosphate buffer including 6 M NaCl (pH 6.5). The precipitated protein (0.2 mL; two aliquots for protein and carbonyl concentration) was then treated with 1 mL of cold trichloroacetic acid (TCA; 10%) and centrifuged (5 min at 5000 rpm). One mL of 2 M HCl was added to one of the shots, and the second was homogenised in 2 M HCl with 0.2% (w/v) 2,4 dinitrophenylhydrazones (DNPH) to estimate the carbonyl content. After 1 h of incubation at 25 °C in the dark, specimens were precipitated. To ensure the elimination of excess DNPH, the percolated samples were washed with 1 mL of ethanol:ethyl acetate (1:1, v/v), and the washing was repeated until the ethanol/ethyl acetate extract became discoloured. The sediments were then mixed with 1.5 mL of guanidine-containing buffer, sodium phosphate. The quantity of carbonyls was expressed as nanomoles of carbonyl per milligram of protein using a protein hydrazone absorption coefficient of 21.0 nM^−1^ cm^−1^ at 370 nm.

##### Sensory Evaluation

There were 24 qualified panellists from the Food Technology Department in the Agriculture faculty at Kafrelshiekh University, ranging in age from 25 to 50 years. The meatball samples were put on a 3-digit-coded covered dish. Colour, chewing, taste, odour, and overall approval of specimens were assessed by the panellists. The sensory assessment was conducted using a 9-point hedonic scale varying from 9 (like extremely) to 1 (dislike extremely) [45].

#### 2.2.5. Statistical Analysis

ANOVA was employed to establish differences between means in the general linear model of SPSS (version 16.0, 2007). For statistical methods, the probability values *p* < 0.05 were deemed significant. All measurements and tests were performed in triplicate.

## 3. Results and Discussion

### 3.1. Characterisation of Microspheres

#### 3.1.1. Size Determination and Sphericity Factor

The size and sphericity factor of CLOs alginate/LPI complex microcapsules were 0.82 ± 0.06 mm and 0.092 ± 0.01, respectively, according to data in Table 1. Since the sphere has the lowest surface/volume ratio of all geometric forms, spherical beads are preferred over irregular shaped beads. Additionally, data in Table 1 revealed that using alginate only as a shell produced microcapsules with a smaller size and sphericity factor than others containing alginate/LPI complex as a shell. The rate of diffusion is related to the rate of the disseminated compound, and hence the surface is engaged in diffusion phenomena. As a result, a smaller sphericity factor indicates a lower surface per volume unit, which means less diffusion of reactive substances (such as oxygen) into the beads, which might change the encapsulated oil [46].

#### 3.1.2. Encapsulation Efficiency

Alginate beads have been criticised for their high gel porosity, which allows loaded bioactive components to diffuse from the gel network into the aqueous medium. Other polymers (such as chitosan) have often been employed in conjunction with sodium alginate to prevent oil loss throughout the formation of alginate beads. The encapsulation efficiency in case of using alginate and alginate/LPI complex as wall materials was 76.49% ± 2.04% and 95.62% ± 1.13%, respectively (Table 1). The inclusion of LPI in our case may have resulted in the formation of a thick network structure having tight voids (pores) that entrapped the CLO droplets within the matrix, resulting in the maximum encapsulation effectiveness [37]. Similar findings were found when different materials such as soy protein [37], gelatin [47], chitosan [48], and starch [49] were added to alginate in order to form a thick network structure and achieve high encapsulation efficiency.

#### 3.1.3. Microscopic Observations

**Figure 1:**
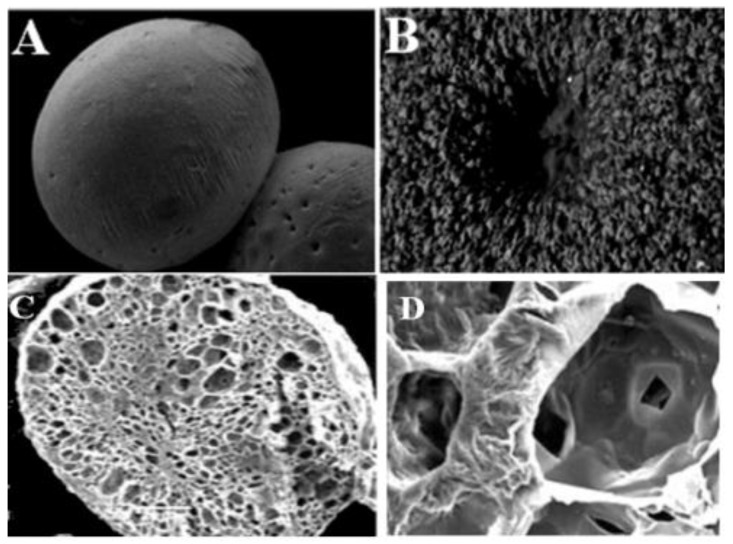
SEM image of alginate/lupin protein isolate complex beads with encapsulated cod liver oil at 150× magnification showing the outer aspect (**A**), the external surface details (**B**), the cross-section (**C**), and the microstructure (**D**).

SEM images of air-dried alginate–LPI complex beads are shown in Figure 1A–D. Figure 1A shows that the formed microcapsules are spherical, which is consistent with their shape indicators (Table 1).

The existence of diverse exterior morphologies (smooth and rough surfaces) was detected as a result of drying and subsequent shrinking, according to surface observations (Figure 1A). Nanocapsules containing oil generated by spray drying showed a similar effect [50]. Several small downturns were seen on the surface of several beads. However, employing greater magnification over the issue zone ruled out the probability of deeper pores (Figure 1B). Our findings were consistent with what was stated by Belščak-Cvitanović et al. [51]. They observed a rough and nonhomogeneous surface of alginate–protein beads, but they delegated the spherical constructions to proteins, stating that morphologies are ascertained by the covalent binding between amide groups of protein and alginate. On the other hand, a cross-section of these beads with encapsulated CLOs (Figure 1C) revealed that they have the structure of a tri-dimensional, porous sponge made of alginate/LPI. The cavity of the cell and its wall can be clearly seen in Figure 1D. In addition, the cell wall wrinkling was visible. The entrapment of CLOs in cells of the alginate/LPI complex matrix is confirmed by this egg-box structural system.

#### 3.1.4. Oil In Vitro Release

**Figure 2:**
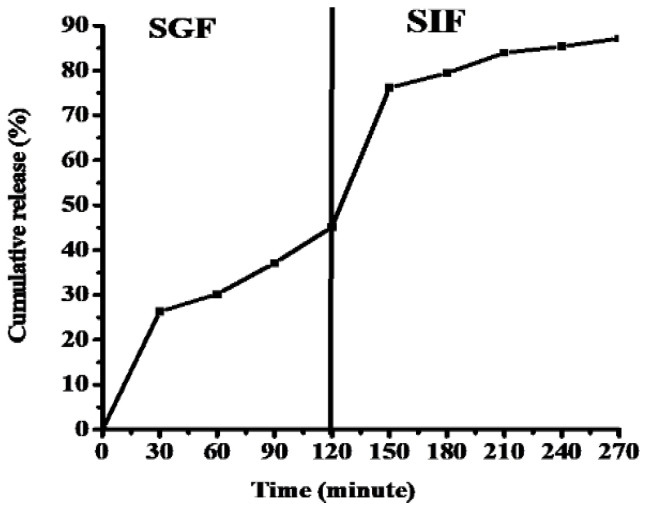
In vitro release of cod liver oil from alginate/lupin protein isolate complex beads in simulated gastric fluid (SGF) and simulated intestinal fluid (SIF).

Figure 2 shows the release characteristics of CLOs from alginate–LPI complex beads into SGF and SIF at 37 °C. The release of CLOs from beads in gastric solution is characterised by a high initial rate in the first 10 min. The quick diffusion of non-encapsulated chemicals from the polymer matrix surface is primarily responsible for the first explosion effect [52]. The proportion of the released CLOs in SGF and SIF was 45.14% and 41.96%, respectively.

At the enteric stage, high oil release is recommended because fats are digested with pancreatic lipase inside the small intestine, and subsequently free fatty acids are taken into the circulation. The modest releases in SGF at pH 1.2 might be attributed to swelling (rehydration) increasing the porousness of the outer layer, and the protease activity assisting in the degradation of the interior matrix. Furthermore, dehydrated sodium alginate formed a porous non-soluble layer known as alginic acid skin [53]. When the microspheres are subjected to SIF at pH 6.8, the alginic acid skin transforms into a soluble layer and viscoelastic structure, allowing for more COL release. These findings are consistent with those of Volić et al. [37], who discovered that alginate–soybean protein isolate complex beads released more thyme oil in SIF than in SGF.

#### 3.1.5. Thermal Stability

Since the sample is processed under controlled settings in which the oxidative process reaches its final phases, the Rancimat accelerated test has acquired acceptability as an indirect indicator of oil stability. Fats are oxidised to produce short-chain volatile components, which are gathered in deionised water and used to increase their conductivity. As a result, longer induction times (IT) indicate greater oxidative stability. The IT for both free and encapsulated CLOs after heat treatment is shown in Table 2.

The IT of encapsulated and free CLOs that were thermally exposed at 70 °C for 24 h was longer than that of CLOs that were thermally exposed at 100 °C for 24 h. Despite the fact that there were no significant changes (*p* < 0.05) in IT values of encapsulated CLOs thermally treated at 70 or 100 °C for 24 h, those thermally treated at 100 °C had shorter ITs (lower oxidative stability) than those thermally treated at 70 °C. In almost any event, IT values for encapsulated oils were substantially longer than free oils, confirming the encapsulating system’s protective impact. Furthermore, the microcapsule wall material may enhance the protection level of unstable oils, especially in the presence of antioxidant components. Encapsulating systems made out of polysaccharides and proteins, according to Gallardo et al. [17], are excellent in linseed oil protection against external conditions. The extra protective impact was attributed to the antioxidant activity created by proteins, according to these researchers. Proteins and peptides from many sources, such as cereals, legumes, microalgae, and animal sources, have been extensively studied for their antioxidant activity [54].

### 3.2. Physicochemical Properties of Meatballs during Storage

#### 3.2.1. pH Value

**Figure 3:**
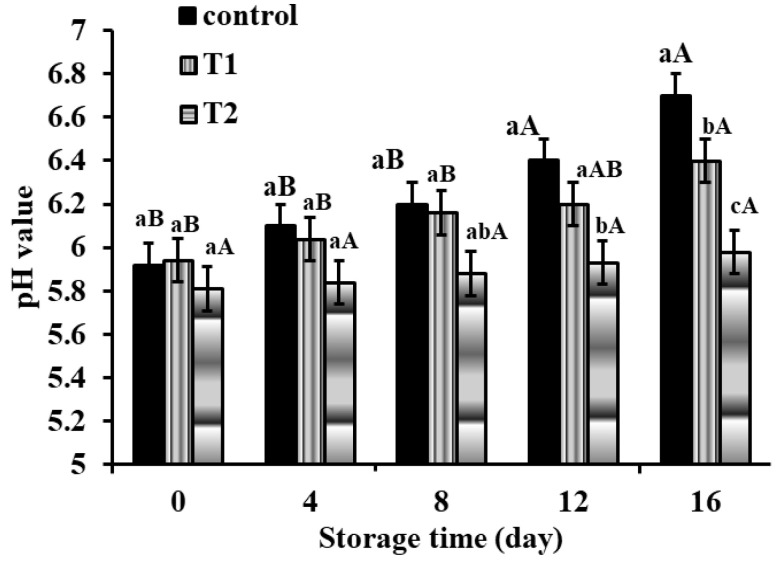
Changes in pH value in meatball samples with incorporated cod liver oil microcapsules during storage at 4 ± 1 °C. Error bars represent standard deviation (n = 3). T1: free cod liver oil direct addition, T2: encapsulated cod liver oil. Different superscripts of (a–c) lowercase letters indicate significant differences at *p* < 0.05 between treatments at each storage time. Different superscripts of (A, B) uppercase letters indicate significant differences at *p* < 0.05 of each sample over the storage period.

The pH value of meat is a key determinant of its quality. The pH of meatball samples was measured throughout the course of 16 days of storage at 4 ± 1 °C. As shown in Figure 3, the control specimen had the highest pH value, whereas the sample with CLOs encapsulated had the lowest. The main pH value in all samples was about 5.89, with this ratio steadily increasing over the storage period, according to the findings. Since the encapsulating procedure protects the CLO from degradation, a significant difference (*p* < 0.05) in pH value was detected after 8 days of storage between the specimens including microcapsules and the control samples. The rise in pH value in control and CLO direct addition specimens relative to those containing microcapsules was verified. This might be attributed to microbial enzymes decomposing nitrogenous substances [5,55].

#### 3.2.2. TBARS Value

**Figure 4:**
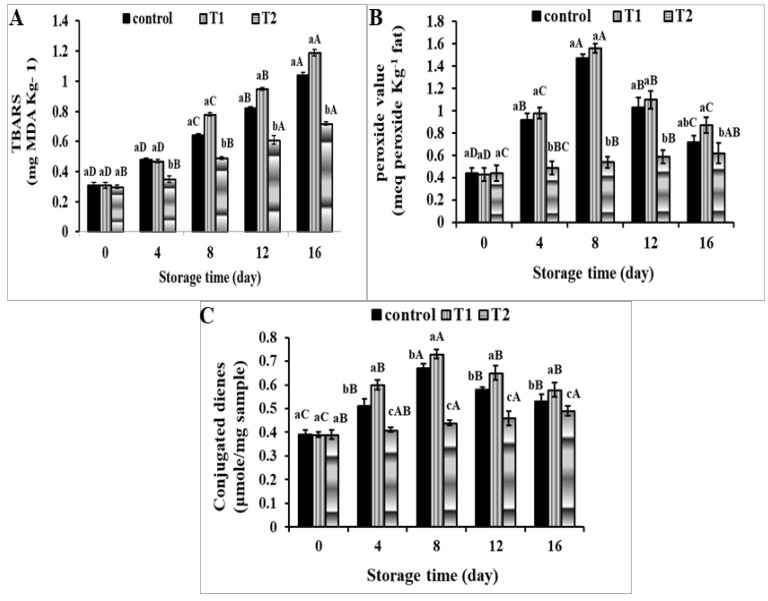
Changes in TBARS (mg MDA Kg ^–^^1^) (**A**), peroxide value (meq peroxide Kg ^–^^1^ fat) (**B**), and conjugated dienes (μmole/mg of sample) (**C**) in meatball samples with incorporated cod liver oil microcapsules during storage at 4 ± 1 °C. Error bars represent standard deviation, (n = 3). T1: free cod liver oil direct addition, T2: encapsulated cod liver oil. Different superscripts of (a–c) lowercase letters indicate significant differences at *p* < 0.05 between treatments at each storage time. Different superscripts of (A, B) uppercase letters indicate significant differences at *p* < 0.05 of each sample over the storage period.

The variations in TBARS content in meatballs enhanced with CLOs after storage at 4 ± 1 °C for 16 days are shown in Figure 4A. At time zero, the control, CLO direct addition, and CLO-encapsulated specimens had TBARS values of 0.31, 0.31, and 0.30 mg MDA kg^−1^, respectively. After 16 days, the CLO direct addition specimens had quickly climbed to 1.19 mg MDA kg^−1^, followed by control (1.04 mg MDA kg^−1^) and CLO-encapsulated (0.72 mg MDA kg^−1^) specimens. This is consistent with prior results in fish oil microcapsule-enriched sausage, and chicken nuggets [56,57]. In the treatments, enriched and non-enriched CLOs, storage of the meatball samples resulted in a considerable rise in the TBARS readings. After 16 days of storage, there was a larger rise in TBARS in the enhanced specimens with CLO direct addition than in the control specimens. The CLO-encapsulated samples had the lowest TBARS value. These findings might be attributed to the microcapsule walls, which operate as a barrier, protecting and minimising CLO and omega-3 PUFA interactions with oxidant agents in meat products [58].

#### 3.2.3. Peroxide Value

The initial PV of all meatball samples was about 0.44 meq kg^−1^, as shown in Figure 4B, but it quickly rose throughout the storage duration of up to 16 days. In both control and CLO direct addition specimens, the PVs reached the threshold limit value on the eighth day of storage, and thereafter rapidly decreased. However, the PV in CLO-encapsulated meatball samples remained below the limit value until the 16th day of cold storage at 4 ± 1 °C. After 16 days of storage, the PVs of the meatball control, CLO direct addition, and CLO-encapsulated specimens were 0.72, 0.87, and 0.62 meq kg^−1^, respectively.

The results showed that until day 8 of storage, control and CLO direct addition samples had a significant lipid oxidation and PVs maximum at the end of the primary auto-oxidation. The PVs were reduced after 8 days, which might be related to hydroperoxide breakdown into secondary lipid oxidation products [59]. The specimens that included CLO microcapsules, on the other hand, had a lower impact on oxidation. It is obvious that microencapsulation protected CLOs and omega-3 polyunsaturated fatty acids in beef from degrading influences. The findings are consistent with those previously published by Solomando et al. [5].

#### 3.2.4. Conjugated Dienes

The variations in conjugated dienes content in meatballs enhanced with CLOs throughout the storage at 4 ± 1 °C for 16 days are shown in Figure 4C. The conjugated dienes content of all meatball samples at time zero was around 0.39 mole/mg of specimen. The conjugated dienes content in the control and CLO direct addition specimens reached the maximal limit value on the eighth day of storage, and then rapidly decreased. However, the conjugated dienes content in CLO-encapsulated meatball samples remained below the limit value until the 16th day of cold storage at 4 ± 1 °C. After 16 days of storage, the conjugated dienes content of the meatball control, CLO direct addition, and CLO-encapsulated specimens was 0.53, 0.58, and 0.49 mole/mg of specimen, respectively. During the storage period, the enriched specimens with CLO direct addition had a higher increase in conjugated dienes than the control specimen.

#### 3.2.5. Carbonyl Content

**Figure 5:**
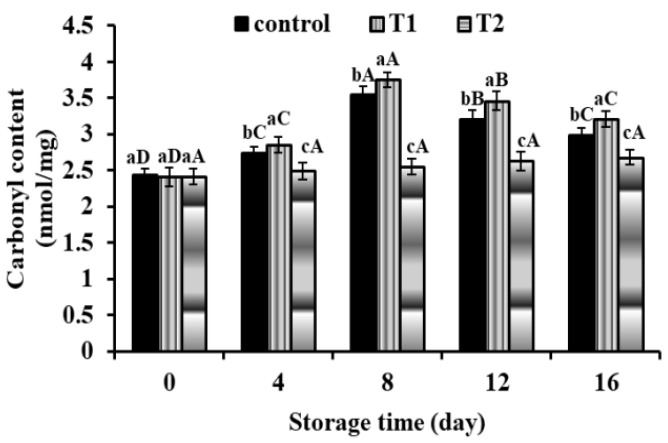
Changes in carbonyl content (nanomoles of carbonyl/milligram of protein) in meatball samples with incorporated cod liver oil microcapsules during storage at 4 ± 1 °C. Error bars represent standard deviation (n = 3). T1: free cod liver oil direct addition, T2: encapsulated cod liver oil. Different superscripts of (a–c) lowercase letters indicate significant differences at *p* < 0.05 between treatments at each storage time. Different superscripts of (A, B) uppercase letters indicate significant differences at *p* < 0.05 of each sample over the storage period.

Carbonyl content is used as a marker for protein oxidation. Carbonyl derivatives may be formed when amino acids are oxidised [60]. The carbonyl content of the meatball samples was also assessed after 16 days of storage at 4 ± 1 °C (Figure 5).

All meatball samples had an initial carbonyl content of 2.41 to 2.43 nmol/mg protein, which significantly rose throughout storage in the CLO direct addition specimen to 3.21 nmol/mg protein, followed by control (2.98 nmol/mg protein) and CLO-encapsulated (2.67 nmol/mg protein) specimens after 16 days. Control and CLO direct addition specimens showed notable lipid oxidation until day 8 of storage, with a carbonyl content peak at the end of the initial auto-oxidation. Carbonyl concentration dropped after 8 days, perhaps owing to the carbonyl conversion to Schiff-base adducts [60]. The carbonyl concentration of enhanced samples with CLO direct addition was considerably greater than that of the control on all storage days (*p* < 0.05). The CLO-encapsulated specimens, on the other hand, had the lowest carbonyl concentration. It is possible that the difference between free CLOs and encapsulated oils is attributable to the creation of more fatty acid oxidation products in this therapy than in treatment including microcapsules. More free radicals may speed up the oxidation of protein in meat [34]. As a consequence of this research, microcapsule formulations may be useful in preserving proteins against oxidation.

#### 3.2.6. Meatballs’ Sensory Evaluation

The mean sensory criteria scores, i.e., colour, odour, taste, chewing, and acceptability of meatball specimens, were statistically analysed for the storage periods until an off-flavour was evident (Table 3). Generally, no significant change (*p* < 0.05) was detected at time zero between all treatments. Nevertheless, it was noted that the colour, odour, taste, chewing, and acceptance values of all prepared meatball specimens reduced with increasing the storage period up to 16 days.

Results indicated that control and CLO direct addition specimens had poor scores in all sensory metrics throughout the storage periods. However, the CLO direct addition specimen produced a significant fishy odour and was discarded in the early stages of storage (day 8) owing to its undesirable colour, odour, taste, and acceptability. The CLO-encapsulated samples showed good scores and acceptability up to day 16 of storage. This is due to the microcapsule walls, which work as a good barrier and prevent the oxidation of CLOs in meat products, as well as the fact that microcapsules may protect omega-3 PUFA from oxidation and radicals within the meat product. The sensory evaluation result could be related to the variations in the oxidation rates, and the storage effect has been deeply noted in control and CLO direct addition specimens, which could be attributed to the high values of pH, TBARS, PVs, conjugated dienes, and carbonyl content [5]. The findings are in accordance with earlier research on enhanced meat products with fish oil microcapsules, i.e., sausages [5] and pork burgers [61]. From the above-mentioned findings, microencapsulation is the ideal technique to improve nutrition, hide off-flavours, and simplify storage, without detrimental impacts on the physical, chemical, or functional aspects of the food items.

## 4. Conclusions

This research investigated the preparation of functional meatballs containing CLO microcapsules. The microcapsules were characterised, as well as the quality characteristics of the enriched meatballs. The CLO microspheres had a uniform round shape, high encapsulation efficiency, and high oil in vitro release in the stimulated gastrointestinal conditions, according to the research results. In addition, when comparing the pH, TBARS, PV, conjugated dienes, and carbonyl content of meatballs supplemented with CLO microcapsules to un-encapsulated and/or control samples, a significant decrease in their values was observed. Meatballs enriched with CLO microcapsules received favourable sensory evaluations, but meatballs directly containing CLOs were rejected (fishy taste). As a result, the viability of alginate/LPI complex microcapsules containing CLOs to improve meat products for thermal stability, oil preservation, and sensory qualities of meatballs could be suggested.

## Figures and Tables

**Table 1:** Characteristics of alginate–lupin protein complex beads with encapsulated cod liver oil microcapsules.

Type of Microcapsules	Size, mm	Sphericity Factor	Encapsulation Efficiency (%)
Alginate–lupin protein complex	0.82 ± 0.06	0.092 ± 0.01	95.62 ± 1.13
Alginate	0.63 ± 0.12	0.073 ± 0.01	76.49 ± 2.04

Data are presented as mean ± SD.

**Table 2:** Effect of thermal treatment in oxidative stability of encapsulated and free cod liver oil.

Thermal Treatment	Induction Time (IT), Hours	
	Encapsulated Oil	Free Oil
70 °C, 24 h	5.62 ± 0.25 ^Aa^	4.40 ± 0.18 ^Ab^
100 °C, 24 h	5.49 ± 0.22 ^Aa^	0.52 ± 0.21 ^Bb^

Data are presented as mean ± SD. Means with different superscripts of (A, B) uppercase letters in a column are significantly different at *p* < 0.05. Means with different superscripts of (a, b) lowercase letters in a row are significantly different at *p* < 0.05.

**Table 3:** Sensory evaluation of cooked meatballs enriched with cod liver oil microcapsules stored at 4 ± 1 °C for 16 days.

Sensory Attributes	Treatments	Storage Days				
		0	4	8	12	16
**Odour**	Control	8.00 ± 0.19 ^Aa^	7.52 ± 0.40 ^Aa^	5.08 ± 0.26 ^Bb^	*R	R
	T1	6.98 ± 0.21 ^Aa^	5.00 ± 0.38 ^Bb^	R	R	R
	T2	8.00 ± 0.20 ^Aa^	7.70 ± 0.41 ^Aa^	7.04 ± 0.31 ^Ab^	6.63 ± 0.25 ^b^	5.80 ± 0.21 ^c^
**Colour**	Control	8.00 ± 0.45 ^Aa^	6.33 ± 0.36 ^Bb^	5.41 ± 0.29 ^Bc^	R	R
	T1	7.75 ± 0.38 ^Aa^	5.00 ± 0.19 ^Cb^	R	R	R
	T2	8.00 ± 0.41 ^Aa^	7.51 ± 0.28 ^Aa^	7.18 ± 0.30 ^Aa^	6.41 ± 0.17 ^b^	6.00 ± 0.24 ^c^
**Taste**	Control	8.00 ± 0.32 ^Aa^	7.15 ± 0.22 ^Ab^	5.94 ± 0.43 ^Bc^	R	R
	T1	5.50 ± 0.29 ^Ba^	5.00 ± 0.31 ^Bb^	R	R	R
	T2	8.00 ± 0.26 ^Aa^	7.24 ± 0.37 ^Aa^	6.51 ± 0.25 ^Ab^	6.13 ± 0.40 ^b^	5.82 ± 0.34 ^c^
**Chewing**	Control	8.00 ± 0.33 ^Aa^	7.19 ± 0.42 ^Ab^	6.00 ± 0.39 ^Bc^	R	R
	T1	5.7 ± 0.44 ^Ba^	5.10 ± 0.36 ^Ba^	R	R	R
	T2	8.00 ± 0.35 ^Aa^	7.38 ± 0.47 ^Aa^	6.92 ± 0.30 ^Ab^	6.07 ± 0.51 ^b^	5.42 ± 0.29 ^c^
**Overall** **acceptance**	Control	8.00 ± 0.19 ^Aa^	7.05 ± 0.26 ^Ab^	5.61 ± 0.14 ^Bc^	R	R
	T1	6.48 ± 0.22 ^Ba^	5.03 ± 0.28 ^Bb^	R	R	R
	T2	8.00 ± 0.30 ^Aa^	7.46 ± 0.33 ^Aa^	6.91 ± 0.28 ^Ab^	6.31 ± 0.41 ^b^	5.76 ± 0.38 ^c^

Data are presented as mean ± SD. T1: free cod liver oil direct addition, T2: encapsulated cod liver oil. Each value was an average of twenty-four replicates. Different superscripts of uppercase letters in a column indicate significant differences at *p* < 0.05 between treatments at each storage time. Different superscripts of lowercase letters in the same row indicate significant differences at *p* < 0.05 in each treatment during storage time. R: reject.

## Data Availability

The data presented in this study are available in this article.
